# Influence of Patient Characteristics on the Effectiveness of Inhaled Nitric Oxide Therapy for Pulmonary Hypertension Following Cardiac Surgery: A Post-marketing Surveillance in Japan

**DOI:** 10.7759/cureus.99788

**Published:** 2025-12-21

**Authors:** Shuhei Doteguchi, Emi Matsugi, Shigeki Takashima, Ryota Kawai, Hisako Yoshida, Ayumi Shintani, Motohiro Okayasu

**Affiliations:** 1 Department of Medical Affairs, Mallinckrodt Pharmaceuticals, Tokyo, JPN; 2 Department of Pharmacovigilance, Mallinckrodt Pharmaceuticals, Tokyo, JPN; 3 Department of Medical Statistics, Osaka Metropolitan University Graduate School of Medicine, Osaka, JPN

**Keywords:** cardiac surgery, inhaled nitric oxide, post-marketing surveillance, postoperative, pulmonary hypertension

## Abstract

Background

A post-marketing surveillance (PMS) of INOflo^®^ for Inhalation 800 ppm in Japan (N = 2,817) established its real-world safety and effectiveness in improving hemodynamic parameters during the perioperative period in patients undergoing cardiac surgery. However, the patient population was heterogeneous with respect to characteristics such as age and baseline hemodynamic parameters.

Objective

The objective of this study is to evaluate patient characteristics associated with the effectiveness of inhaled nitric oxide (iNO) for pulmonary hypertension following cardiac surgery in Japan.

Methods

This study was a retrospective analysis of the PMS data of INOflo. Adults aged ≥15 years with a mean pulmonary artery pressure (mPAP) ≥20 mmHg at baseline (i.e., prior to iNO initiation) who commenced iNO following cardiac surgery were included. The PMS prospectively monitored the safety and effectiveness of INOflo in Japan. The primary endpoint was the change in mPAP from baseline to 24 hours after iNO initiation. Multivariable regression analysis explored patient characteristics (age, BMI, sex, and prior pulmonary vasodilator medication) associated with the primary endpoint.

Results

A total of 427 Japanese patients were included (median (IQR) age: 71.0 (60.0-77.0) years). Significant improvements in hemodynamic parameters were observed, including mPAP at 24 and 48 hours from baseline. No patient characteristics were significantly associated with the change in mPAP from baseline to 24 hours after iNO initiation. However, patients aged 30 years or with a BMI of 35 did not show significant changes in mPAP, whereas other age and BMI categories demonstrated statistically significant improvements.

Conclusions

iNO treatment was associated with improvements in hemodynamics in patients with pulmonary hypertension following cardiac surgery, irrespective of patient characteristics such as age, BMI, sex, or prior pulmonary vasodilator therapy in this analysis. Conversely, the models suggest that differences in age and BMI may influence the magnitude of iNO effectiveness on the change in mPAP.

## Introduction

Inhaled nitric oxide (iNO) relaxes pulmonary vascular smooth muscle [1] and reduces mean pulmonary artery pressure (mPAP) and pulmonary vascular resistance [2,3], contributing to hemodynamic improvement in pulmonary hypertension. In 2015, INOflo^®^ for Inhalation 800 ppm (INOflo), a medicinal gas containing NO 800 ppm (Mallinckrodt Pharmaceuticals, Tokyo, Japan), was conditionally approved by the Japanese Ministry of Health, Labour and Welfare for the improvement of perioperative pulmonary hypertension associated with cardiac surgery [4-6]. Approval was largely based on a small phase three study conducted in 12 pediatric and six adult Japanese patients with pulmonary hypertension during cardiac surgery [5,7] but was contingent upon further post-marketing surveillance (PMS) being conducted [8].

The PMS of INOflo included 2,817 patients in Japan and established the real-world safety of INOflo, including treatment-related adverse events, as well as its effectiveness in improving hemodynamic parameters during the perioperative period of cardiac surgery in both pediatric and adult patients [8]. However, the PMS was conducted in accordance with the Japanese Pharmaceuticals and Medical Devices Act and did not account for differences in patient background. As such, the analysis population was heterogeneous with respect to clinical characteristics such as age, BMI, sex, and baseline hemodynamic values, all of which may influence the response to iNO. In addition, the timing of iNO initiation differed among patients, with some receiving it before or during surgery, further contributing to variability in baseline status. Although the study reported an overall improvement in mPAP in adult patients, it did not explore how patient background may have contributed to variability in treatment effect. While several reports have described inadequate hemodynamic responses to iNO in some patients [9-11], the underlying reasons for this remain unclear. Therefore, further analysis of patient characteristics associated with treatment efficacy using data from Japanese clinical settings is warranted.

In 2022, the European Society of Cardiology/European Respiratory Society revised their guidelines to lower the threshold to mPAP >20 mmHg for patients with pulmonary hypertension [12]. Although the package insert of INOflo does not define specific hemodynamic parameters for initiating therapy or assessing treatment response in perioperative pulmonary hypertension associated with cardiac surgery, the effect of iNO may be more appropriately evaluated by focusing on patients with elevated mPAP who meet the guideline-based definition of pulmonary hypertension [1]. Therefore, the primary objective of this study was to analyze the relationship between patient characteristics and the change in mPAP before and after iNO in patients with pulmonary hypertension whose baseline mPAP was ≥20 mmHg following cardiac surgery, based on PMS data of INOflo in Japan.

## Materials and methods

Study design and participants

Full details regarding the PMS study design and eligibility have been published previously [8]. Briefly, this was a post hoc analysis using data from the PMS, which prospectively monitored the safety and effectiveness of INOflo following its conditional approval for perioperative pulmonary hypertension associated with cardiac surgery in pediatric and adult patients. The PMS protocol did not define the timing of iNO initiation, cardiac surgery procedures, or the use of other medications and devices (such as artificial ventilation or mechanical circulatory support), as the surveillance was observational and noninterventional. Data from eligible patients at 253 clinical sites in Japan were collected through anonymized registration forms submitted to a central registration system. The surveillance commenced in November 2015, and data for eligible patients were analyzed until the target sample size was reached in December 2020.

For this analysis, adults aged ≥15 years were eligible if they had an mPAP ≥20 mmHg prior to iNO initiation and had undergone cardiac surgery, regardless of the time elapsed since the procedure. Patients were excluded if they met any of the following criteria: (1) baseline clinical information was not available within 48 hours prior to iNO initiation or (2) they died on the same day that iNO treatment was started. The first criterion was introduced to minimize the influence of longer intervals between baseline assessment and iNO initiation. The second criterion was applied to avoid confounding related to terminal clinical status, which could hinder proper evaluation of iNO effectiveness. Inclusion of such cases may have biased the observed association between iNO administration and clinical outcomes.

Outcome measures

Clinical laboratory data were collected during the surveillance at baseline (i.e., before iNO initiation, with timing not predefined), at the time of iNO initiation, and at one to four, 24, and 48 hours after iNO initiation, at treatment completion or withdrawal (prior to weaning), and ≤48 hours after completion/withdrawal.

The primary endpoint of this analysis was the change in mPAP from baseline to 24 hours after iNO initiation. Secondary endpoints included the change in mPAP, partial pressure of arterial oxygen/fraction of inspired oxygen (PaO₂/FiO₂), cardiac index (CI), and mean pulmonary arterial pressure/mean systemic blood pressure (Pp/Ps) from baseline to 24 and 48 hours after iNO initiation. For each parameter, differences between baseline and each post-initiation time point were calculated.

Statistical analysis

Patient background and clinical characteristics were summarized using descriptive statistics, including medians and IQRs for continuous variables and frequencies and proportions for categorical variables.

To assess the time trajectory of changes in mPAP, CI, and Pp/Ps from baseline to 24 and 48 hours after iNO initiation, mixed-effects models were fitted to estimate the mean of each endpoint as a function of time (five time points: baseline, iNO initiation, one to four hours, 24 hours, and 48 hours after iNO initiation), with time included as a categorical variable. These models were adjusted for age, sex, BMI, and prior medications (pulmonary vasodilators), with covariates selected a priori based on clinical relevance. Additionally, regression analyses were performed using restricted cubic spline functions with three knots for age and BMI. Linear contrasts (linear combinations of regression parameters) were computed to compare baseline values with those obtained 24 and 48 hours after iNO initiation [13].

In subgroup analyses, the time trajectory of changes in PaO₂/FiO₂ was assessed separately based on baseline PaO₂/FiO₂ values (≤200, >200 and ≤400, >400) to maintain consistency with previous reports [14].

To analyze the association between the change in mPAP and each hemodynamic parameter (mPAP, PaO₂/FiO₂, CI, and Pp/Ps) at baseline, linear regression models were used. The change in mPAP from baseline to 24 hours after iNO initiation served as the dependent variable, and each hemodynamic parameter was treated as an explanatory variable with adjustment for age, sex, BMI, and pulmonary vasodilators. These regression analyses also used restricted cubic spline functions with three knots for hemodynamic parameters, age, and BMI. Each hemodynamic parameter was included separately in the regression model to avoid multicollinearity.

For the analysis of associations between change in mPAP from baseline to 24 hours after iNO initiation and patient characteristics (age, sex, BMI, and use of pulmonary vasodilators), mixed-effects models were fitted with mPAP as the dependent variable and time since iNO administration (as a categorical variable) as an explanatory variable. To evaluate effect modification, a cross-product term between time point (baseline, iNO initiation, one to four hours, 24 hours, and 48 hours after iNO initiation) and each patient characteristic was added separately to each model. Age and BMI were modeled as continuous covariates, and nonlinear interaction terms were incorporated using restricted cubic spline functions with three knots to more accurately assess potential effect modification without discarding information. The change in mPAP from baseline to 24 hours after iNO initiation was evaluated using linear contrasts, comparing estimated means at baseline with those at 24 hours across age categories (30-80 years in 10-year intervals) and BMI categories (15-35 kg/m² in 5 kg/m² intervals) [13].

Missing values in dependent and explanatory variables were imputed using multiple imputation with predictive mean matching. Five imputed datasets were created and combined using Rubin’s rules [15]. All P values were two-tailed, with a significance level of α = 0.05 indicating statistical significance. All statistical analyses were performed using R statistical software version 4.3.1 (R Foundation for Statistical Computing, Vienna, Austria).

## Results

Study population

**Figure 1:**
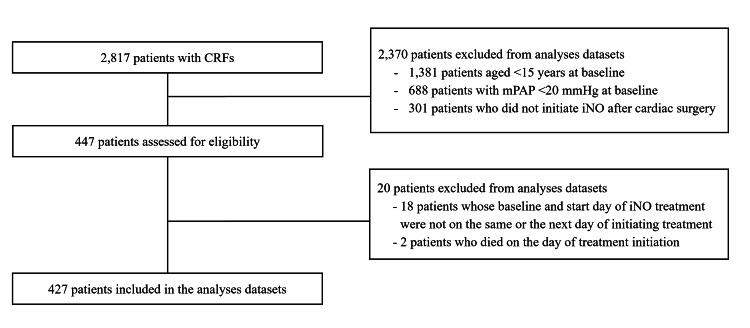
Patient flow diagram CRF, case report form; iNO, inhaled nitric oxide; mPAP, mean pulmonary artery pressure

Case report forms were collected for 2,817 registered patients who received iNO (Figure 1), of whom 447 met the inclusion criteria. Twenty patients were excluded from the analysis. Reasons for exclusion included patients whose baseline and post-administration dates did not occur on the same day or the day after iNO initiation (n = 18) and patients whose date of death coincided with the date of iNO initiation (n = 2). Therefore, a total of 427 patients were included in the analysis.

Patient background characteristics and prior pulmonary vasodilator medications are summarized in Table 1. The median (IQR) age was 71.0 (60.0-77.0) years. There was a slightly higher proportion of males (57.8%) than females (42.2%), and the median (IQR) BMI was 22.9 (20.3-25.6) kg/m². Most patients had not received pretreatment with pulmonary vasodilators (Table 1). Pulmonary vasodilators that were used in at least one patient included ambrisentan (n = 2 (0.5%)) and tadalafil (n = 3 (0.7%)).

**Table 1:** Patient clinical characteristics CI, cardiac index; mPAP, mean pulmonary artery pressure; mSBP, mean systemic blood pressure; PaO₂/FiO₂, partial pressure of arterial oxygen/fraction of inspired oxygen; Pp/Ps, mean pulmonary arterial pressure/mean systemic blood pressure; Q1, first quartile; Q3, third quartile

Parameter	N = 427
Age (years)	
Median (Q1, Q3)	71.0 (60.0-77.0)
Age category (years), n (%)	
≤30	8 (1.9)
>30-≤40	19 (4.4)
>40-≤50	39 (9.1)
>50-≤60	43 (10.1)
>60-≤70	104 (24.4)
>70-≤80	149 (34.9)
>80	65 (15.2)
Sex, n (%)	
Male	247 (57.8)
Female	180 (42.2)
BMI (kg/m²)	
Median (Q1, Q3)	22.9 (20.3-25.6)
Number of missing	4
BMI category (kg/m²), n (%)	
<15	7 (1.6)
≥15-<20	91 (21.3)
≥20-<25	199 (46.6)
≥25-<30	99 (23.2)
≥30	27 (6.3)
Missing	4 (0.9)
Pulmonary vasodilators	
Yes	6 (1.4)
No	421 (98.6)
mSBP (mmHg)	
Median (Q1, Q3)	100.0 (88.0-118.0)
Number of missing	16
mPAP (mmHg)	
Median (Q1, Q3)	28.0 (24.0-32.0)
Number of missing	0
PaO₂/FiO₂	
Median (Q1, Q3)	222.2 (126.8-355.3)
Number of missing	68
CI (L/min)	
Median (Q1, Q3)	3.6 (2.6-4.6)
Number of missing	184
Pp/Ps	
Median (Q1, Q3)	0.4 (0.3-0.5)
Number of missing	16

Outcomes

**Figure 2:**
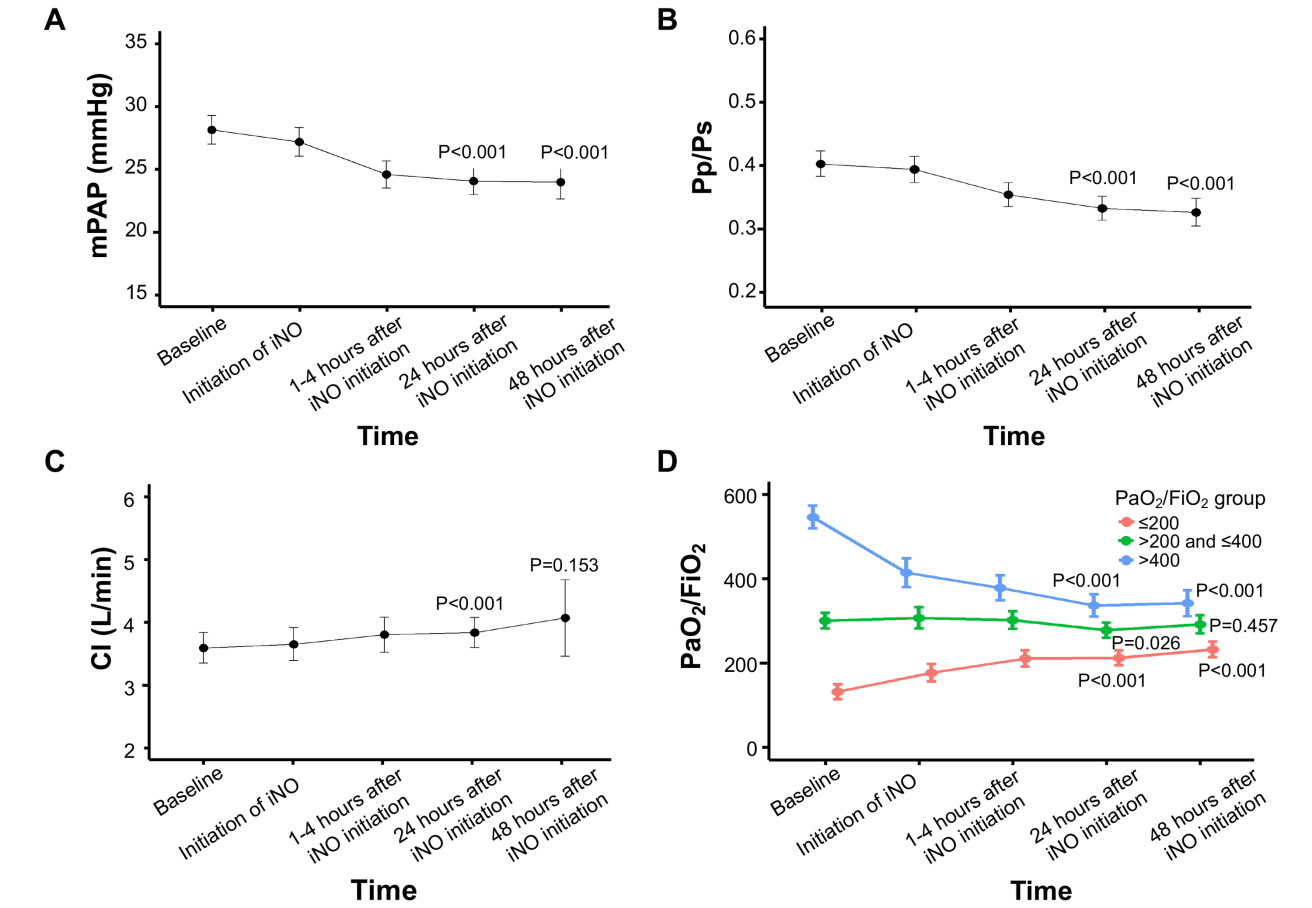
Change in hemodynamic parameters from baseline to 24 and 48 hours after iNO initiation, including (A) mPAP, (B) Pp/Ps, (C) CI, and (D) PaO₂/FiO₂ The P values represent comparisons between baseline and 24 hours and between baseline and 48 hours after iNO initiation. CI, cardiac index; iNO, inhaled nitric oxide; mPAP, mean pulmonary artery pressure; PaO₂/FiO₂, partial pressure of arterial oxygen/fraction of inspired oxygen; Pp/Ps, mean pulmonary arterial pressure/mean systemic blood pressure

With respect to the primary endpoint, a significant decrease in mPAP was observed 24 hours after iNO initiation compared with baseline (Figure 2A). A significant reduction in mPAP was also observed at 48 hours. The mean change in mPAP from baseline was -4.07 mmHg (95% CI -4.73, -3.41) at 24 hours and -4.16 mmHg (95% CI -5.14, -3.18) at 48 hours after iNO initiation. Regarding secondary endpoints, iNO treatment was associated with significant improvements at 24 and 48 hours in hemodynamic parameters, including Pp/Ps, CI (at 24 hours only), and PaO₂/FiO₂ (in the PaO₂/FiO₂ ≤200 group) (Figure 2B-2D).

**Figure 3:**
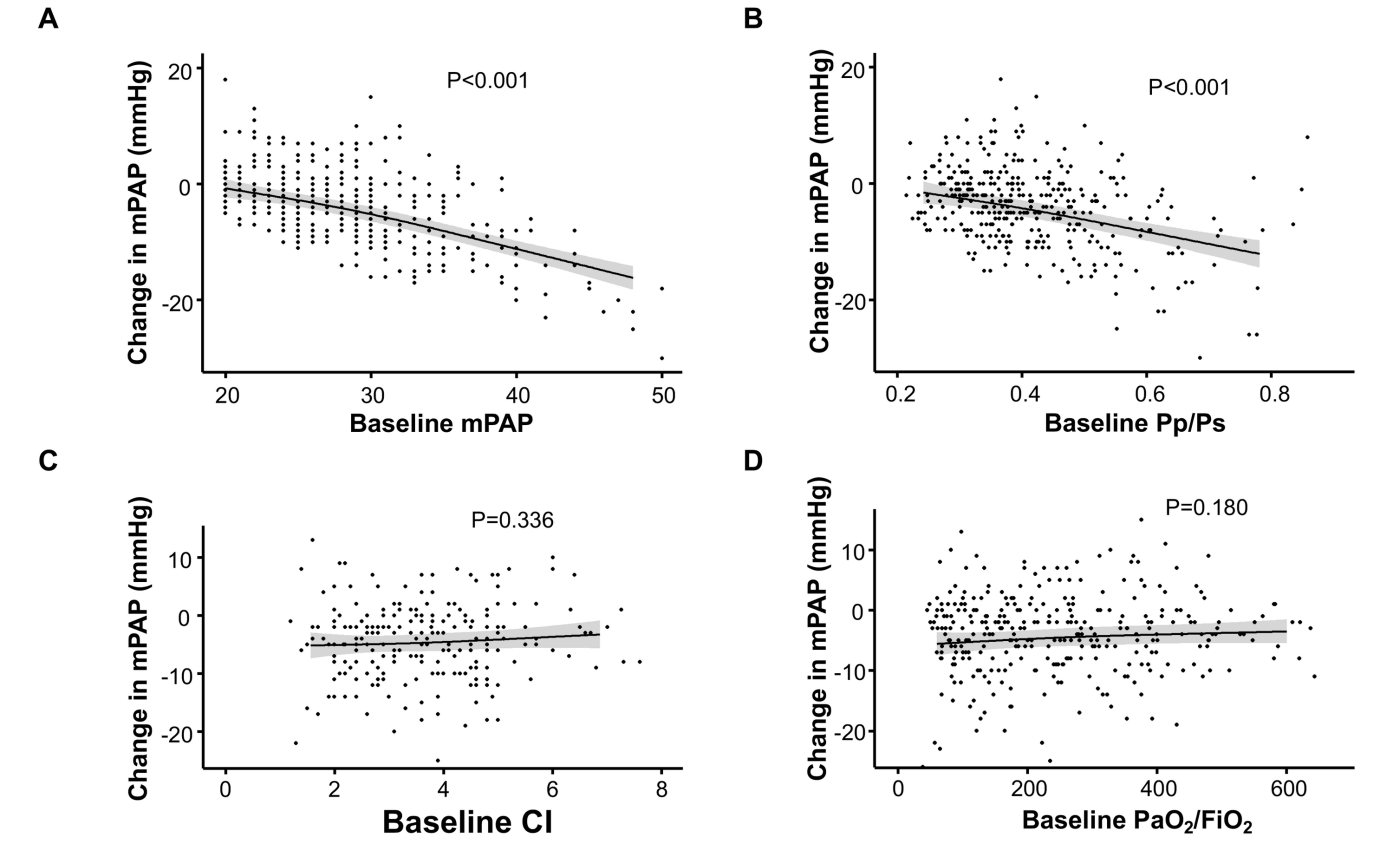
Correlation between the change in mPAP from baseline to 24 hours after iNO initiation and (A) mPAP, (B) Pp/Ps, (C) CI, and (D) PaO₂/FiO₂ The P values indicate whether the slope of each parameter is significantly different from zero; a P value < 0.05 suggests a significant linear association with the change in mPAP from baseline to 24 hours after iNO initiation. CI, cardiac index; iNO, inhaled nitric oxide; mPAP, mean pulmonary arterial pressure; PaO₂/FiO₂, partial pressure of arterial oxygen/fraction of inspired oxygen; Pp/Ps, mean pulmonary arterial pressure/mean systemic blood pressure

Compared with baseline, significant improvements in PaO₂/FiO₂ were observed at 24 hours (P < 0.001) and 48 hours (P < 0.001) after iNO treatment in patients in the ≤200 group. In contrast, PaO₂/FiO₂ remained relatively unchanged in patients in the >200 to ≤400 group at 24 hours (P = 0.026) and 48 hours (P = 0.457) after iNO treatment and was significantly decreased at 24 hours (P < 0.001) and 48 hours (P < 0.001) in patients in the >400 group. According to multivariable regression analyses, there was a significant correlation between the change in mPAP from baseline to 24 hours after iNO initiation and baseline mPAP and Pp/Ps (P < 0.001 each) (Figure 3A, 3B). There was no significant correlation between the change in mPAP and baseline CI (P = 0.336) or baseline PaO₂/FiO₂ (P = 0.180) (Figure 3C, 3D).

**Figure 4:**
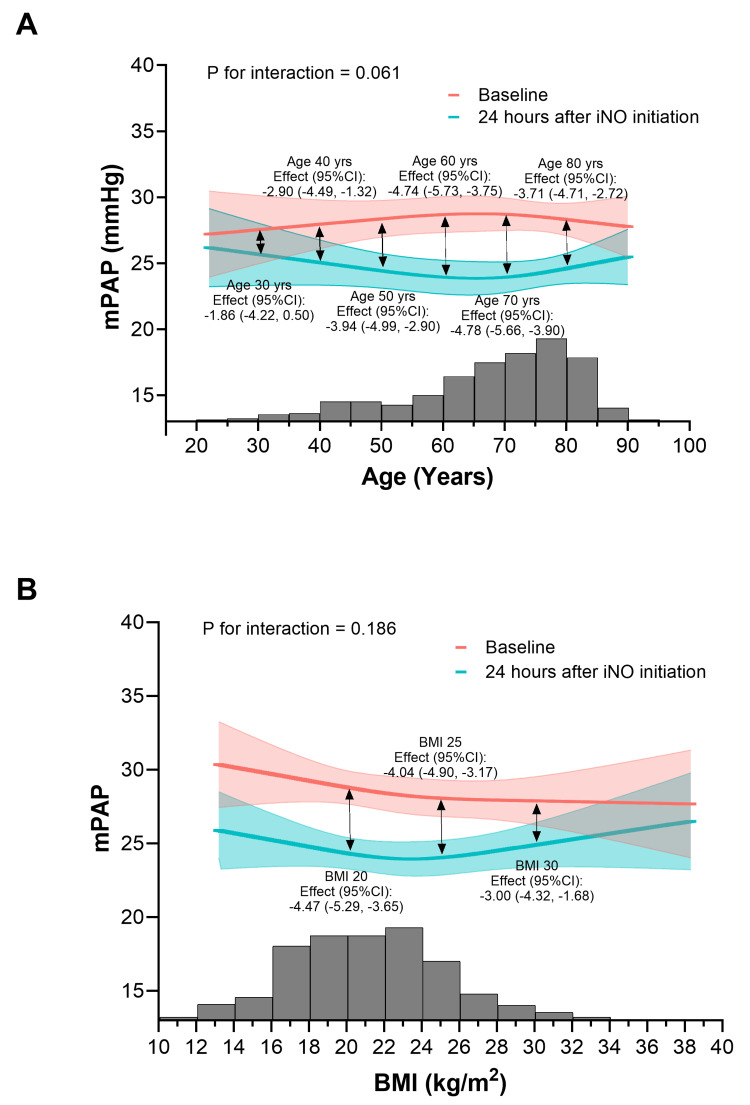
Correlation between the change in mPAP from baseline to 24 hours after iNO initiation and patient characteristics, including (A) age and (B) BMI To assess the correlation between patient characteristics and changes in mPAP, expected mPAP values from the mixed-effects model were plotted on the vertical axis, and patient characteristics were plotted on the horizontal axis. Histograms show the distribution of age (five-year intervals, 20-24 to 96-100 years) and BMI (2-kg/m² categories, 10-12 to 46-48 kg/m²). P values for interaction indicate whether the effect of iNO on mPAP varied according to age or BMI. Clinically meaningful P values of interaction suggest that the change in mPAP is modified by the respective variable. iNO, inhaled nitric oxide; mPAP, mean pulmonary artery pressure

No patient characteristics showed significant effect modification or interaction with the change in mPAP from baseline to 24 hours after iNO initiation with respect to age, sex, BMI, or prior medications (pulmonary vasodilators) (Figure 4). However, no significant change in mPAP was observed in patients aged 30 years (Figure 4A), whereas other age categories showed statistically significant changes. Similarly, patients with a BMI of 35 showed reduced changes in mPAP, while other BMI categories demonstrated statistically significant improvements (Figure 4B), indicating a diminished effect in this subgroup. Sex was not associated with changes in mPAP (P for interaction = 0.593), and iNO significantly reduced mPAP regardless of sex. Likewise, there was no significant interaction based on prior use of pulmonary vasodilators (P for interaction = 0.164). A significant decrease in mPAP was observed both with and without pulmonary vasodilators, although baseline mPAP tended to be higher in patients receiving pulmonary vasodilators (P < 0.001 and P = 0.005, respectively).

## Discussion

This analysis included 427 patients initiating iNO treatment for pulmonary hypertension following cardiac surgery from a large PMS in Japan (N = 2,817). The data provide valuable insights into the influence of patient characteristics on the effectiveness of iNO treatment with respect to improvements in hemodynamic parameters, including change in mPAP (primary endpoint) for pulmonary hypertension, defined as an mPAP ≥20 mmHg [13]. Consistent with the broader results reported by Matsugi et al. (2023) [8], significant improvements in hemodynamic parameters were observed, including mPAP, PaO₂/FiO₂ (PaO₂/FiO₂ ≤200 group), CI, and Pp/Ps, in patients receiving iNO following cardiac surgery.

Importantly, no patient characteristics were found to have a significant influence on change in mPAP after iNO initiation with respect to age, sex, BMI, or prior pulmonary vasodilator medication. These results suggest that these characteristics are generally not associated with changes in mPAP. However, considering a P value threshold for interaction of <0.20, as used in another study assessing adiposity and chronic kidney disease [16], clinically meaningful interactions may exist between age, BMI, prior pulmonary vasodilator use, and change in mPAP after iNO initiation. Different trends regarding the effect on mPAP were observed depending on age or BMI. For example, both baseline mPAP and the amount of change in mPAP were higher with increasing age, peaking in patients aged 60-70 years. This finding is consistent with prior reports showing that pulmonary artery systolic pressure at rest and during exercise increases with age, likely due to reduced vascular compliance and/or cardiac remodeling [17-19]. Furthermore, experimental and human studies have demonstrated that constitutive production of endogenous NO decreases with aging, potentially due to age-related comorbidities [20], suggesting that iNO may compensate for these deficiencies and explain the greater change in mPAP observed in this population.

Conversely, there was a trend toward a smaller change in mPAP in patients with a BMI above 25. Obesity has been associated with certain postoperative pulmonary complications, longer mechanical ventilation, and prolonged ICU stay [21,22]. In obese patients with pulmonary hypertension, proinflammatory mediators may promote remodeling of the pulmonary vasculature [23]. These characteristics may therefore influence iNO effectiveness, highlighting the need for further studies evaluating iNO treatment in obese patients.

Regarding changes in mPAP by age and BMI, no significant change was observed in adults aged 30 years or in patients with a BMI of 35. Although the analysis included a limited number of younger patients and those with BMI >30, these results suggest a diminished effect of iNO treatment in these subgroups. For younger patients, lower baseline mPAP compared with other subgroups may have contributed to the smaller change. Additionally, in contrast to older patients, younger individuals may have sufficient endogenous NO production, limiting the observable effect of iNO [20]. Further studies with larger sample sizes are warranted to confirm the influence of these patient characteristics on mPAP changes.

In this analysis, significant changes were also observed in Pp/Ps, which correlated with baseline mPAP. Pp/Ps has been reported as an indicator for evaluating iNO effectiveness in the perioperative period of cardiac surgery, including valve procedures and congenital heart disease [24,25]. Another study showed that mean systemic blood pressure/mPAP after induction of anesthesia could predict hemodynamic complications after cardiac surgery [26]. These findings suggest that monitoring parameters such as Pp/Ps, in addition to mPAP, may provide a more comprehensive evaluation of iNO effectiveness.

The effectiveness of iNO on oxygenation varied depending on baseline PaO₂/FiO₂. Improvements were greatest in patients with PaO₂/FiO₂ ≤200 at baseline, classified as Berlin-defined moderate-to-severe respiratory failure [14]. In contrast, patients with higher baseline PaO₂/FiO₂ showed decreased oxygenation, particularly those with PaO₂/FiO₂ >400, who exhibited a marked decline from baseline to 48 hours. However, the clinical impact of this reduction is likely limited, as baseline oxygenation in these patients exceeded the threshold for even mild acute respiratory distress syndrome [14]. Consequently, the correlation between changes in mPAP and baseline PaO₂/FiO₂ was weak. While potential confounders, such as ventilator use, extracorporeal membrane oxygenation, and timing of iNO initiation following cardiopulmonary bypass, should be considered, these findings suggest that mPAP reduction may, in some cases, occur independently of changes in oxygenation.

In the most recent ESC/ERS guidelines, the mPAP threshold for defining pulmonary hypertension was lowered from ≥25 to >20 mmHg [13]. Although the approved indication for INOflo does not specify a threshold [5], our analysis showed that iNO treatment was effective in patients with pulmonary hypertension, including those with modestly elevated mPAP (≥20 mmHg). Additionally, baseline mPAP correlated with the magnitude of mPAP reduction after iNO initiation. A rat model study suggested that iNO effects on pulmonary artery dynamics differ between control and pulmonary hypertension models [27], indicating that the degree of pulmonary hypertension may influence iNO responsiveness.

These results must be interpreted considering the study’s limitations. First, as a PMS and post hoc analysis, data limitations may have influenced multivariable regression results. Potential confounders include artificial ventilation, mechanical circulatory support, timing of cardiopulmonary bypass withdrawal, other medications (inotropes and diuretics), surgical procedure and technique, and primary disease or comorbidities. For example, patients reoperated after congenital heart disease with complex hemodynamics (including Fontan circulation) could not be separately analyzed. Second, although multiple imputation with predictive mean matching addressed missing values, careful interpretation of CI and PaO₂/FiO₂ is warranted due to incomplete data. Definitions for fatal cases could not be applied because the timing of death was unknown. Third, PMS data reflect real-world practice across multiple sites, introducing potential selection bias and variability in parameter monitoring. The absence of a comparator arm may also limit interpretation. Finally, pulmonary vascular resistance and pulmonary capillary wedge pressure could not be analyzed due to insufficient data, despite their relevance to the iNO mechanism of action.

## Conclusions

In this analysis of patients initiating iNO treatment for pulmonary hypertension following cardiac surgery using data from a PMS of INOflo in Japan, iNO treatment was associated with improvements in mPAP regardless of patient characteristics such as age, BMI, sex, or prior pulmonary vasodilator use, although some limitations should be considered. These findings support the use of iNO for postoperative management of pulmonary hypertension in real-world clinical practice in Japan. Conversely, our models suggest that age and BMI may influence the magnitude of mPAP change, indicating potential variability in iNO effectiveness across different subgroups. These exploratory results highlight the need for future studies with enriched populations to more clearly identify patient characteristics that may predict clinical outcomes with iNO treatment.
